# Quality of Co-Prescribing NSAID and Gastroprotective Medications for Elders in The Netherlands and Its Association with the Electronic Medical Record

**DOI:** 10.1371/journal.pone.0129515

**Published:** 2015-06-25

**Authors:** Dedan Opondo, Stefan Visscher, Saeid Eslami, Robert A. Verheij, Joke C. Korevaar, Ameen Abu-Hanna

**Affiliations:** 1 Department of Medical Informatics, Academic Medical Center, Amsterdam, The Netherlands; 2 Netherlands Institute for Health Services Research (NIVEL), Utrecht, The Netherlands; Karolinska Institutet, ITALY

## Abstract

**Objective:**

To assess guideline adherence of co-prescribing NSAID and gastroprotective medications for elders in general practice over time, and investigate its potential association with the electronic medical record (EMR) system brand used.

**Methods:**

We included patients 65 years and older who received NSAIDs between 2005 and 2010. Prescription data were extracted from EMR systems of GP practices participating in the Dutch NIVEL Primary Care Database. We calculated the proportion of NSAID prescriptions with co-prescription of gastroprotective medication for each GP practice at intervals of three months. Association between proportion of gastroprotection, brand of electronic medical record (EMR), and type of GP practice were explored. Temporal trends in proportion of gastroprotection between electronic medical records systems were analyzed using a random effects linear regression model.

**Results:**

We included 91,521 patient visits with NSAID prescriptions from 77 general practices between 2005 and 2010. Overall proportion of NSAID prescriptions to the elderly with co-prescription of gastroprotective medication was 43%. Mean proportion of gastroprotection increased from 27% (CI 25–29%) in the first quarter of 2005 with a rate of 1.2% every 3 months to 55%(CI 52–58%) at the end of 2010. Brand of EMR and type of GP practice were independently associated with co-prescription of gastroprotection.

**Conclusion:**

Although prescription of gastroprotective medications to elderly patients who receive NSAIDs increased in The Netherlands, they are not co-prescribed in about half of the indicated cases. Brand of EMR system is associated with differences in prescription of gastroprotective medication. Optimal design and utilization of EMRs is a potential area of intervention to improve quality of prescription.

## Introduction

Pain is a common problem among elderly persons living in the community as well as in institutions of organized care such as nursing homes[1][2]. Tsai *et al*. reported a 50% prevalence of pain among community dwelling elderly patients[3] and a 65% prevalence among nursing home residents in Taiwan[4]. Therefore the consumption of pain medication among the elderly is high. Population studies in the US have shown that 70% of people older than 65 years use non-steroidal antiinflammatory drugs (NSAIDs) at least once per week while about 34% use the drugs at least once a day[5].

Many studies have shown an increased risk of gastrointestinal complications among NSAID users, particularly peptic ulcers and its attendant complications such as upper gastrointestinal bleeding and perforations[6]. Laine *et al*. reported a prevalence of 15–30% of peptic ulcers and an annual prevalence of 1.0–1.5% of upper gastrointestinal bleeding among NSAID users[7].

Adherence to safe NSAID prescribing practices as proposed by clinical guidelines has been shown to reduce upper gastrointestinal toxicities[8]. For example, patients using NSAIDs and gastroprotection with proton pump inhibitors have a lower risk of upper gastrointestinal toxicities compared to patients without gastroprotective medication risk of 1.8 versus 1.1[9].

Clinical guidelines on safe NSAID prescribing include the Assessing Care of Vulnerable Elders (ACOVE) clinical rule recommending a concomitant gastroprotective medication, proton pump inhibitors or misoprostol, to elderly patients at high risk of upper gastrointestinal bleeding[10,11]. This clinical rule has been adopted by a team of experts in geriatric care for use in general practice in the Netherlands[12]. Similarly, the Dutch College of General Practitioners also recommends the prescription of gastroprotective medication to elderly patients at high risk for upper gastrointestinal events. In 2009, this has been corroborated by a report in The Netherlands, from an expert group with a focus on optimizing extramural medication safety, with specific recommendations for prescribing PPIs in regular NSAID users with an increased risk of GI complications[13]. Previous studies have indicated that sub-optimal and inappropriate prescription of medications exist in primary care settings despite various interventions which have been implemented to improve the quality of prescribing[14].

Safe prescription practices in primary care may be affected by factors including the electronic medical record (EMR) system in use. EMR system types, with six main types in the Netherlands, may use different approaches, such as reminders and alerts, to support medication prescription. We hypothesize that differences in EMR systems used in the Netherlands contribute to difference in coprescription of NSAID with gastroprotective medications.

In this study, we assessed the proportion of co-prescribing NSAIDs and gastroprotective medications, and investigated its association with the EMR system type in general practice in the Netherlands between 2005 and 2010 based on the Dutch translation of the ACOVE clinical indicators[12].

## Methods

We extracted prescription data for all NSAIDs and gastroprotective medications between 1-1-2005 and 31-12-2010 from EMR systems of GP practices participating in the Dutch NIVEL Primary Care Database. The NIVEL Primary Care Database started in 1992 as the Netherlands Information Network of General practice (LINH) and developed into a multidisciplinary primary care database in recent years, encompassing not only data from GP practices but also from out of hours services, psychologists and other primary care disciplines. In the Netherlands, all citizens are registered with a GP practice and GPs act as a gatekeeper for further access to specialized care. During the study period about 90 general practices participated, with a total practice population of 350,000 patients. The database encompasses consultation claims data, health problems, lab test results, prescriptions and referrals, patient age and gender, practice type and EMR system brand.

We note that all EMRs are designed according to standards developed by the Dutch College of General Practitioners. By design any brand of EMR can be used in any type of GP practice. The EMRs are web-based and a doctor within a practice has his or her separate log in credentials.

Prescriptions are recorded in general practice using the Anatomical Therapeutic Classification (ATC) developed by the World Health Organization (WHO). Topical preparations of NSAIDs were excluded from the data because they have limited adverse effects on the gastrointestinal system. Gastroprotective agents (GPAs) included all proton pump inhibitors and misoprostol containing preparations. **Table 1**shows the Non steroidal anti-inflammatory medications which we included in the analysis. The prescription data from the general practices included in this study are contained in S1 Dataset that accompanies this article.

**Table 1:** List of Non Steroidal Anti-inflammatory Drugs (NSAIDs) included in the study grouped by pharmacological class.

Pharmacological class	ATC class code	Name	ATC code
Acetic acid derivative	M01AB	Indomethacin	M01AB01
		Aceclofenac	M01AB16
		Diclofenac combinations	M01AB55
		Sulindac	M01AB02
		Diclofenac	M01AB05
Butylpyrazolidine	M01AA	Phenylbutazone	M01AA01
Coxibs	M01AH	Valdecoxib	M01AH03
		Rofecoxib	M01AH02
		Celecoxib	M01AH01
		Etoricoxib	M01AH05
Fenamate	M01AG	Tolfenamic	M01AG02
Oxicam	M01AC	Tenoxicam	M01AC02
		Meloxicam	M01AC06
		Piroxicam	M01AC01
Propionic acid derivatives	M01AE	Dexibuprofen	M01AE14
		Tiaprofenic acid	M01AE11
		Flurbiprofen	M01AE09
		Ketoprofen	M01AE03
		Naproxen	M01AE02
		Ibuprofen	M01AE01
Others	M01AX01	Nabumetone	M01AX01

### Measurements and outcome variable

The primary outcome variable was the proportion of NSAIDs prescriptions with concomitant co-prescription of gastroprotective medication. Co-prescription was measured as the concurrence of records of NSAID and gastroprotective medication within a 24 hours time frame This proportion was calculated by dividing the number of NSAID prescriptions with a concomitant gastroprotective medication by the total number of NSAID prescriptions. Proportion of gastroprotection was aggregated for every general practice per quarter of a year, i.e. 3 months. The first quarter was the period from 1st January 2005 to 31st March 2005 while the 24th quarter was the period from 1st October 2010 to 31st December 2010.

The following variables were included in the analysis: type of practice and brand of Electronic Medical Record system used in the general practice. All six EMR systems brands were included in the study. Four practice types were distinguished, single handed, duo practice (2 GPs), group practice and health centers. Type of general practice and brand of Electronic Medical Record system were treated as categorical data while the proportion of gastroprotection was treated as a continuous variable.

In the Netherlands, there is no need to obtain consent when only registry data obtained from routine care and without patient identifying information are used, as is stated in the selection criteria for the Medical Research Involving Human Subjects Act (WMO)[15].

### Analysis

Univariate and multivariate linear regression analysis was performed to explore associations between gastroprotection and the variables brand of EMR and type of practice.

Random effects linear regression analysis was used to model trends in prescription of gastroprotective medications from 2005 to 2010 based on the six brands of EMR systems. The random intercept was modeled to represent each brand of EMR at the beginning of the study period while the random slope represented the rate of change of gastroprotection for each brand of EMR. This means that prescriptions were clustered by the brand of electronic medical record system. Temporal profiles of gastroprotection for each brand of EMR system were estimated from the model and presented graphically.

## Results

A total of 91,521 patient visits with NSAID prescriptions from 77 general practices between January 2005 and December 2010 were included. **Table 2**shows the description of the general practices contributing data to this study. Forty three out of the 77 practices (56%) had a single practitioner while 7 (9%) were organized as health centers. Six different brands of electronic medical record (EMR) systems were used by the GP practices during the study period. None of the GPs changed EMR brand during the study period. **Table 3**shows the distribution of EMRs within the different types of GP practices. A chi-square test of the association between practice type and distribution of EMR was statistically insignificant with a p-value of 0.117.

**Table 2:** Characteristics of general practices and determinants of coprescription of gastroprotective medication with NSAIDS.

Brand of Electronic Medical Record system	Number of practices
EMR 1	11
EMR 2	8
EMR 3	18
EMR 4	13
EMR 5	21
EMR 6	6
**Type of practice**	
Single handed	43
Duo (Two GPs)	16
Group	11
Health Center	7

**Table 3:** Distribution of electronic medical records (EMR) system according to type of general practices in the Netherlands.

	Solo	Duo	Group	Health Centre	TOTAL
EMR 1	2	4	3	2	**11**
EMR 2	6	2	0	0	**8**
EMR 3	11	3	2	2	**18**
EMR 4	11	2	0	0	**13**
EMR 5	11	4	3	3	**21**
EMR 6	2	1	3	0	**6**
**TOTAL**	**43**	**16**	**11**	**7**	**77**

The overall proportion of gastroprotection co-prescription with NSAIDS during this five year period was 43.0%. The overall rate of gastroprotection in all the practices increased from 26.6% (CI 24.6–28.7) in the first quarter of 2005 with 1.2% (CI 1.03–1.3) every 3 months to 54.7% (CI 51.9–57.5%) at the end of 2010.

**Fig 1:**
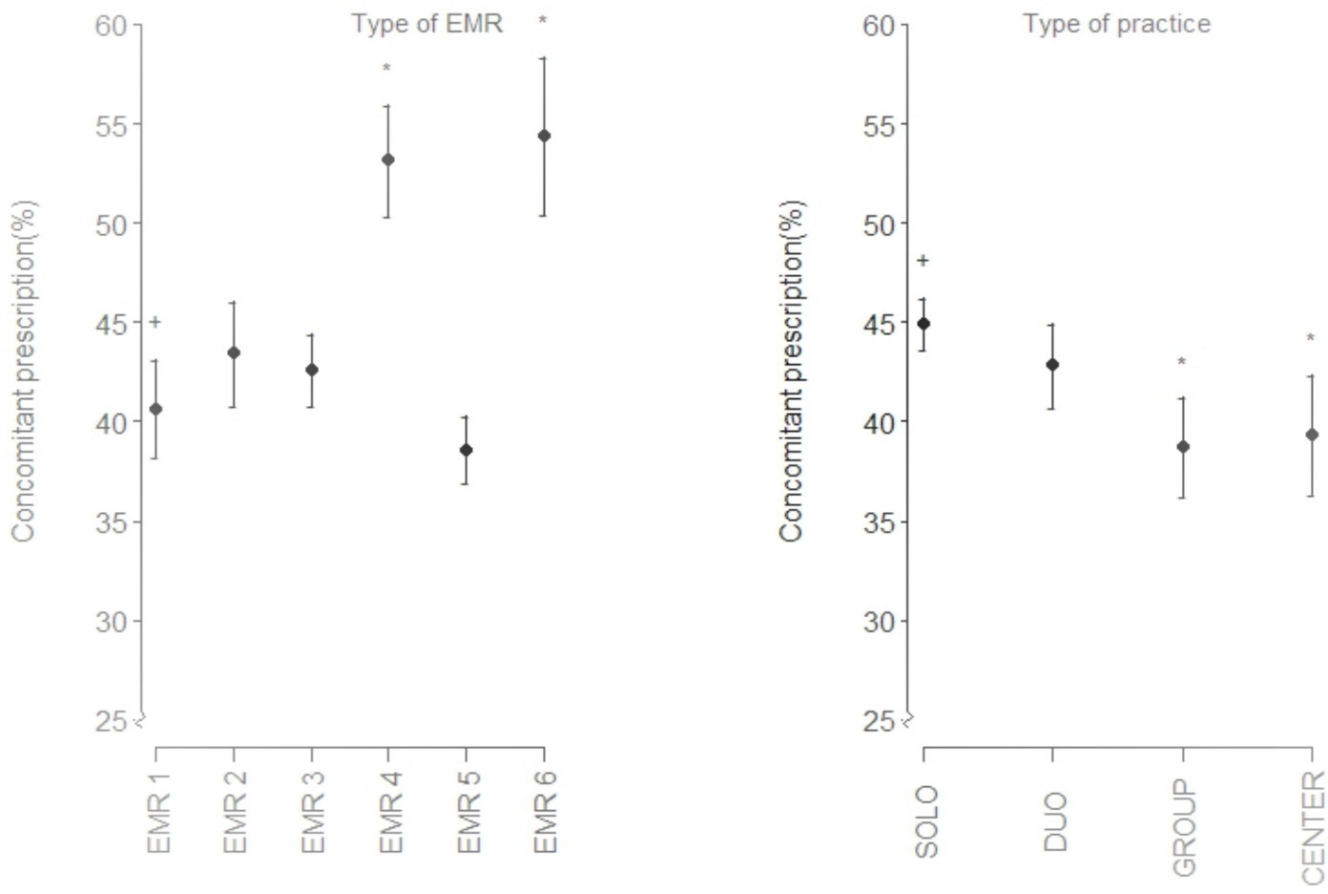
Univariate analysis of proportions of concomitant gastroprotection with NSAIDS based on brand of electronic medical record system and type of general practice. +—reference group in univariate analysis, *—statistically significant different from the reference group, SOLO- A single practitioner’ practice, DUO–A two practitioners’ practice, GROUP- A more than two practitioners’ practice, CENTER- A health center, usually with more primary health care services EMR–Electronic Medical Record System.

Fig 1. shows the mean proportion of concomitant gastroprotection with NSAIDS comparing the brand of EMR system and type of general practice. General practices that used EMR brand 4 and EMR brand 6 had statistically significant higher proportions of prescription of gastroprotective medications compared to EMR brands 1, 2, 3 and 5.

Multivariate linear regression analysis of time, brand of EMR system and type of GP practice showed significant differences in the rate of concomitant gastroprotection as shown in **Table 4**. Statistically significant differences in proportions of gastroprotection were observed with EMR brand 4 and EMR brand 6 compared to EMR brand 1 while the difference in gastroprotection was not significant between EMR brands 2, 3 and 5 as compared to EMR brand 1.

**Table 4:** Regression analysis of determinants of rate of concomitant coprescription of gastroprotective medications with NSAIDs.

	Univariate analysis			Multivariate analysis		
Factors	Coefficient (%)	95% CI	P value	Coefficient (%)	95% CI	P value
Time (quarter)	1.2	1.0–1.3	<0.001*	1.0	0.9–1.1	<0.001*
**Brand of EMR**						
EMR 1–reference	*-*	*-*	*-*	*-*	*-*	*-*
EMR 2	2.8	-0.8–6.3	0.156	1.82	5.1–12.9	0.306
EMR 3	2.0	-1.1–5.0	0.222	1.31	0.1–6.3	0.372
EMR 4	12.5	8.8–16.2	<0.001*	7.15	12.1–20.4	<0.001*
EMR 5	-2.1	-5.0–0.9	0.198	-2.42	-2.6–3.6	0.087
EMR 6	13.7	9.1–18.4	<0.001*	10.32	8.2–17.4	<0.001*
**Type of practice**						
Solo practice-reference	*-*	*-*		*-*	*-*	*-*
Duo practice	-2.1	-4.6–0.4	0.125	-1.7	-3.3–1.1	0.133
Group practice	-6.2	-9.0-(-3.4)	<0.001*	-5.4	-4.8–1.0	<0.001*
Health Center	-5.6	-8.8-(-2.3)	<0.001*	-2.71	-10.5-(-4.4)	0.084

**LL**- Lower limit of confidence interval.

**UL**-Upper limit of confidence interval.

* Statistically significant difference

Group practices had lower rates of gastroprotection compared to single practitioner practices (p-value <0.001). The differences between dual practitioner and group practices, and single practitioner practices were not statistically significant.

**Fig 2:**
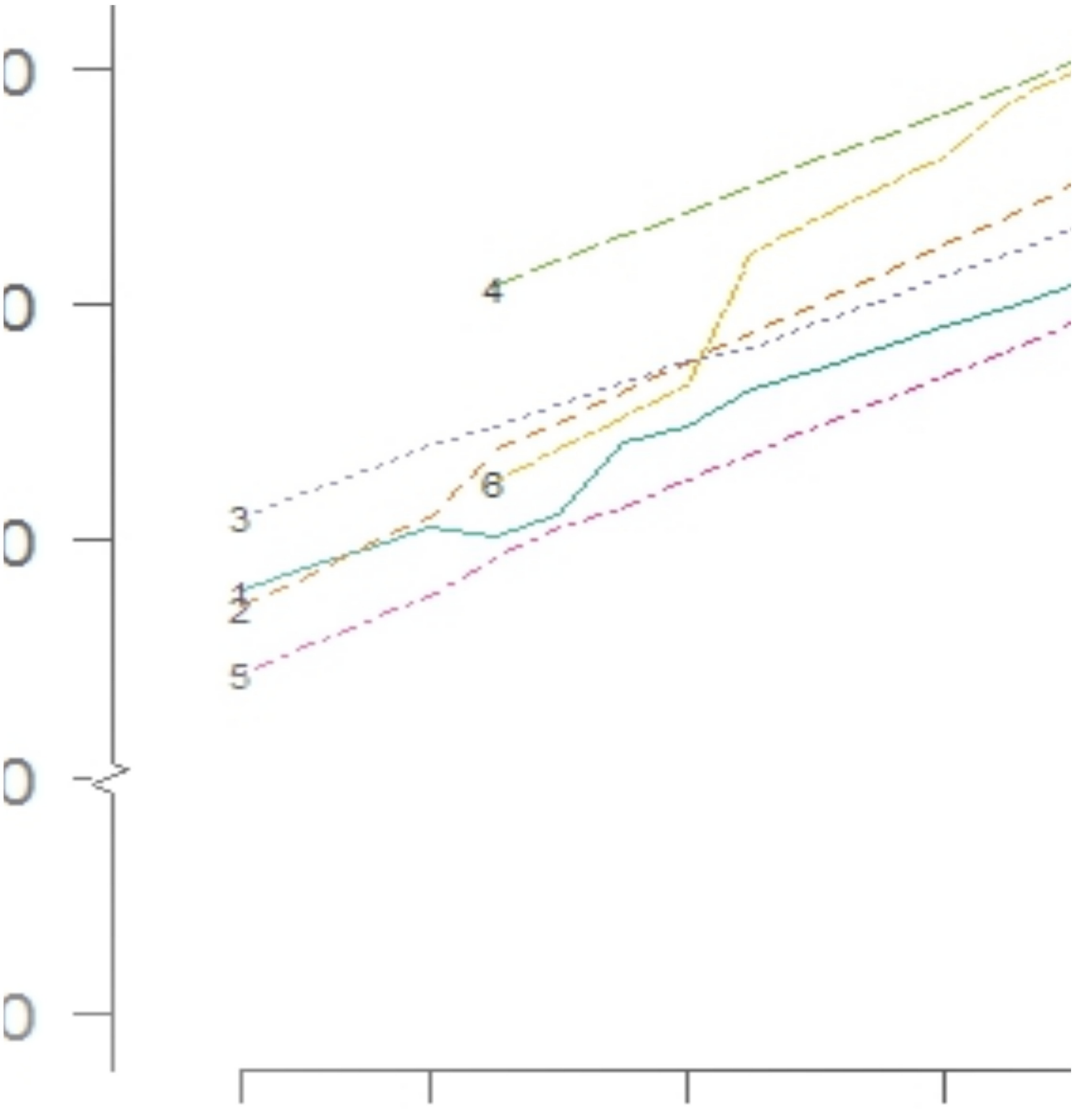
Time trends of the rate of concomitant prescription of gastroprotective medication with NSAIDs between various electronic medical record (EMR) systems.

Fig 2. shows the differences in the rates of prescription of gastroprotective medication over time. EMR brand 4 and brand 6 have a higher mean rate of prescription of gastroprotective medication compared to EMR brands 2, 3 1 and 5 respectively having adjusted for the effect of time. EMR brand 6 showed the highest increase in gastroprotection rates over time.

## Discussion

The mean proportion of concomitant gastroprotection for elderly persons who received NSAIDs in the Netherlands between 2005 and 2010 was 43.0%. Despite the increase of this proportion from 26.6% with a rate of 1.2% every 3 months to 54.7%, gastroprotection is not co-prescribed in about half of the indicated cases. Finally, the Electronic Medical Record system used was associated with the proportion of the concomitant gastroprotection prescription.

Thiefin *et al*. observed that 39% of elderly patients received gastroprotection in a study conducted between June and August 2006 France [12] while a study conducted in Sweden by Fastbom *et al*. found a gastroprotection rate of 22% [13]. Sturkenboom *et al* showed that a majority of patients with one or more gastrointestinal risk factors do not receive appropriate prescription of NSAID and gastroprotective medication or COX-2 selective NSAID[16]. Hartnell et al also found underutilization of gastroprotection for seniors[17]. Similar to our finding of the increase in proportion of concomitant gastroprotection for elderly persons receiving NSAIDs, Valkhoff *et al*. showed an increase from 6.9% to 39.4% over a 10 year period in a study that examined the quality of prescription of gastroprotective medications in one region of the Netherlands from 1996 to 2006 [14]. Lana et al found a 75.8% rate of gastroprotection in primary care centers in Spain[18]. The increase in co-prescription of gastroprotective medication is perhaps due to increasing awareness among physicians about the risk of upper GI bleeding among the elderly patients.

To the best of our knowledge the association between the Electronic Medical Record system with the concomitant prescription of gastroprotection identified in our study is new. Differences in EMR system design may confer advantages to users leading to better quality of prescription. Computerized order entry systems (CPOEs) with inbuilt decision support systems have been shown to improve the quality of medication prescription [15]. All the six EMRs included in this study could generate decision support for prescription of NSAIDs and gastroprotective medications. The six systems allow the GPs to adjust the settings of decision support delivered. Unfortunately, we do not have data that shows the status of decision support for each prescription that was made via the EMRs. Additional insight into the specific functionalities and implementation of the EMRs at the point of care is necessary to fully interpret the differences observed in our study. Unfortunately, a detailed examination of the EMRs as implemented and used during the period of this study is not possible since we conducted a retrospective study. Our identified association is not necessarily causal, and since we did not consider all potential confounders. Future studies should investigate this relationship by appropriately designed trials

Our study has different strengths. First is the large sample with a good representation of the Dutch population. Second, we measured quality of prescribing and explored the variation in the quality of gastroprotection according to the brand of EMR used to issue the prescription. Studies that describe only the overall prescription proportions or trends of care alone do not identify factors that are associated with quality of care. Third, our study also explores the effect of time in the improvement of the quality of prescription. Of note are the gains in quality with time in comparison to the baseline performance. A new intervention would be deemed beneficial only if it can confer benefits that are more than those benefits accruable to time alone.

There are some limitations of our study. First, the ACOVE guidelines are not exactly the same as the current Dutch guidelines. The ACOVE guideline uses 65 years to identify elderly patients who require gastroprotection while the Dutch guidelines suggest that gastroprotection should be initiated at the age of 70 years for all elderly persons and only for high risk elderly persons between 65–70 years. Nevertheless we believe that our study captures the general patterns of co-prescription of gastroprotective agents for high risk elderly persons. Second, the estimate of gastroprotection study was based on the presence or absence of appropriate proton pump inhibitor or misoprostol on the day of NSAID prescription, which is a limited definition of concomitant prescription. Some patients may be carrying current prescription of gastroprotective medication thereby contributing to apparent under prescribing. Similarly some patients may receive refills of gastroprotective medications because they still have stock of NSAIDs at home. The ideal estimate of gastroprotection would be obtained by calculating the dosages and duration of both NSAIDs and gastroprotective medications administered. Third, our dataset did not capture all confounders to correct for relationship between specific EMR and co-prescription of gastroprotective medication. For instance, we did not have data which would indicate whether a specific prescription was made with or without the facilitation of decision support in the EMR.

Future studies need to explore the duration of coverage of gastroprotective agents by investigating the medication dosage duration of treatment and different definitions of elders at “high risk” of gastrointestinal complications. Furthermore, changes in the prevalence of upper gastrointestinal complications related to NSAIDs among the elderly population merit investigation. Finally, future trials should be conducted to explore the design characteristics and utilization of EMR systems that may be associated with higher or lower quality of prescription of gastroprotective medications.

This study demonstrates differences in the percentage of gastroprotection between brands of EMR in the Netherlands. The differences observed suggest the need for interventions to reduce the disparities in the quality of gastroprotection prescription among these systems. Specific policies need to be formulated to identify areas of need for targeted interventions. Developers of GP information systems have a potential opportunity for leveraging the use of clinical decision support systems to contribute to the improvement of quality of prescription to the elderly.

## Conclusion

The proportion of prescription of gastroprotective medications to elderly who receive NSAIDs steadily improved in the Netherlands between 2005 and 2010, but gastroprotection is still not co-prescribed in about half of the indicated cases. The type of GP information system is a modifiable factor associated with concomitant medication. Optimal design and utilization of GP information systems is a potential area of intervention to improve the proportion of gastroprotection prescription in combination with NSAIDs in the elderly.

## Supporting Information

S1 Dataset Prescriptions of NSAIDs and gastroprotective medication in Dutch general practices according to electronic medical records. (XLS)
